# First Characterization of The Venom from *Apis mellifera syriaca*, A Honeybee from The Middle East Region

**DOI:** 10.3390/toxins11040191

**Published:** 2019-03-30

**Authors:** Jacinthe Frangieh, Yahya Salma, Katia Haddad, Cesar Mattei, Christian Legros, Ziad Fajloun, Dany El Obeid

**Affiliations:** 1 Laboratory of Applied Biotechnology (LBA3B), Azm Center for Research in Biotechnology and its Applications, EDST, Lebanese University, Tripoli 1300, Lebanon; jacynthefrangieh@gmail.com (J.F.); yahyasalma@ul.edu.lb (Y.S.); 2 Faculty of Sciences 3, Lebanese University, Michel Slayman Tripoli Campus, Ras Maska 1352, Lebanon; khaddad@ul.edu.lb; 3 Mitochondrial and Cardiovascular Pathophysiology – MITOVASC, Team 2, Cardiovascular Mechanotransduction, UMR CNRS 6015, INSERM U1083, Angers University, 49045 Angers, France; cesar.mattei@univ-angers.fr (C.M.); christian.legros@univ-angers.fr (C.L.); 4 Faculty of Agriculture & Veterinary Sciences, Lebanese University, Dekwaneh, Beirut 2832, Lebanon

**Keywords:** *Apis mellifera syriaca*, bee venom, melittin, LC-ESI-MS, solid phase extraction, in vitro effects

## Abstract

Bee venom is a mixture of several components with proven therapeutic benefits, among which are anti-inflammatory, analgesic, and various cardiovascular conditions. In this work, we analyzed for the first time the proteomic content and biological properties of the crude venom from *Apis mellifera syriaca*, a honeybee from the Middle East region. Using high-performance liquid chromatography-tandem mass spectrometry, we evidence the venom contains phospholipase A2, hyaluronidase, mast cell-degranulating peptide, adolapin, apamin, and melittin. The latter was purified by solid phase extraction method (SPE) and tested in parallel with crude venom for biological activities. Precisely, crude venom—but not melittin—exhibited antibacterial activity against *Staphylococcus aureus* and *Pseudomonas aeruginosa* strains. Alongside, hemolytic activity was observed in human blood subjected to the venom at high doses. *A. mellifera syriaca* venom displayed antioxidant activities, and not surprisingly, PLA2 catalytic activity. Eventually, the venom proved to exert antiproliferative effects against MCF-7 and 3T3 cancer cells lines. This first report of a new bee venom opens new avenues for therapeutic uses of bee venoms.

## 1. Introduction

Bees use their venom as a defense tool against predators, intruders, and for colony defense [1]. Bee venom (BV) is a complex mixture of peptidyl toxins, enzymes, and other trace components, with a wide spectrum of biological activities such as anti-microbial, anti-cancerous, and antioxidant activities [2,3]. It has been used as a therapeutic tool in oriental medicine to treat several human inflammatory diseases such as rheumatism, arthritis, and to relieve back pain [4,5,6]. These medical claims have now found evidence in numerous studies showing that the use of BVs is not restricted to a single therapeutic area, but can be used for different conditions with various pathophysiological substrates, including for the nervous system, for immunity, or for the cardiovascular system [7]. The bioactive compounds of BV termed apitoxin can be divided into (i) proteins such as melittin, apamin, MCD-peptide (mast cell degranulating peptide) and adolapin, (ii) enzymes like phospholipase A2 (PLA2), hyaluronidase, α-glucosidase, acid phosphomonoesterase, and lysophopholipase, and (iii) also amino acids, phospholipids and volatile compounds [2]. Melittin, one of the major BV components, triggers the toxicity of the venom. It has pore-forming activity in the cell phospholipid bilayer, inducing membrane rupture [8]. Several studies have shown that melittin has a broad spectrum of biological, pharmacological, and toxicological activities including anti-bacterial, anti-viral, anti-inflammatory, and anti-tumor properties, together with hemolytic properties [8]. Apamin, another important peptide in BV, is the smallest venom-derived neurotoxin that blocks small-conductance Ca^2+^-activated K^+^ channels (SKCa) [9]. It exerts therapeutic benefits in mouse models of Parkinson disease [10]. Moreover, PLA2, the major enzyme present in BV, has the ability to cleave membrane phospholipids at the sn-2 position to release fatty acids, especially arachidonic acid and lysophospholipid. The arachidonic acid released is a precursor of eicosanoids such as prostaglandins and leukotrienes, which participate in the inflammatory reaction [11]. Then, BVs appear to harbor a large diversity of natural compounds which, as a mixture, contribute to the whole toxicity of the venom but, as single actors, could be used for their pharmaceutical properties [7]. The search for novel activities in BVs is then an attractive way of discovering future natural drugs for a variety of human pathologies [12].

**Figure 1:**
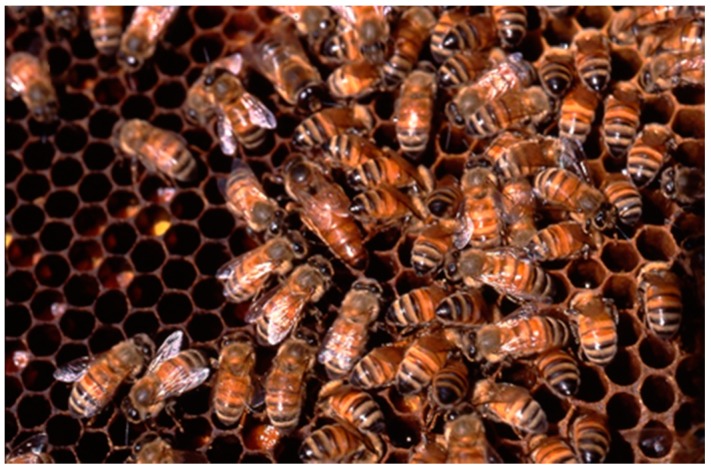
*A. mellifera syriaca* (copyright Dani El Obeid).

*Apis mellifera syriaca* (Figure 1) is the endemic honey bee subspecies present in the Middle East (Lebanon, Syria, Jordan, Palestine, Iraq). Several ecological features make it an interesting venomous animal to study, and a valuable resource to use in agriculture. First, it has a good adaptation to the Mediterranean hot and dry climate [13]. Second, *A. mellifera syriaca*, when compared to the European species, exhibits a higher degree of pest and pathogen resistance, and efficient honey production [14]. But it is threatened by the arrival of commercial breeder lines which could alter its genetic advantages, which should lead to an import restriction or ban on foreign honey bee races [15]. To date, its venom has never been studied.

The aim of this work was to study and analyze for the first time the protein content and biological properties of the venom of *A. mellifera syriaca* (Figure 1). To separate the components of the venom, we performed two different extraction methods. First, the crude venom of *A. mellifera syriaca* was analyzed by LC-ESI-MS in order to identify and characterize the different components of this venom. Second, the SPE (solid phase extraction) method was used to separate the protein constituents from the venom. Eventually, the antibacterial, hemolytic, antioxidant, PLA2, and cytotoxic activities of the crude venom and its major component, melittin, extracted by SPE, were tested. Our work provides the first chemical and biological characterization of a new hymenoptera species.

## 2. Results

### 2.1. Separation and Analysis of Venom Compounds by LC-ESI-MS

**Figure 2:**
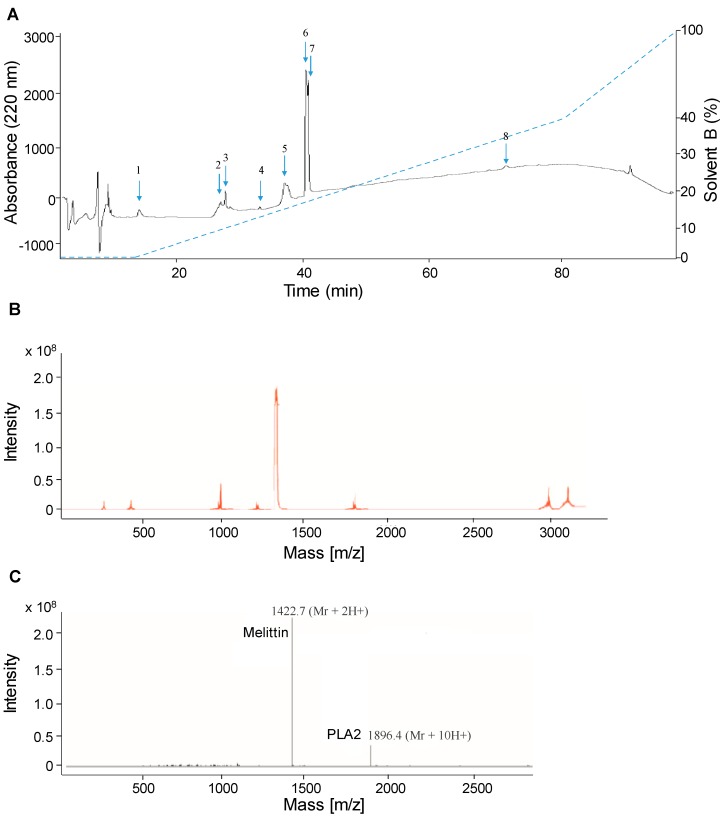
Fractionation of *A. mellifera syriaca* venom using LC-ESI-MS. (**A**) HPLC chromatogram showing reverse-phase C18 fractionation of the venom. (**B**) MS profile of the venom. (**C**) MS profile showing the peaks containing melittin and PLA2.

To separate the venom components, we used two different strategies. First, the venom was eluted using liquid chromatography/electrospray ionization mass spectrometry (LC-ESI-MS). After precipitation in acetonitrile, the venom was further separated by a reverse-phase column (C18). Eight peaks corresponding to distinct proteic components of the *A. mellifera syriaca* venom extract at different retention times appear on the chromatogram (Figure 2A). Each peak corresponds to the elution of a single molecule of the venom, and its abundance is proportional to its intensity (absorbance at 220 nm). The venom eluent was subjected to online coupled ESI-MS analysis to measure the molecular weights and therefore identify all components eluted.

The mass spectrum (Figure 2B) from the venom chromatogram exhibited different peaks (Table S1), among which apamin (2 027 Da), melittin (2 846.4 Da), PLA2 (18 964 Da), MCD-peptide (2 599.8 Da), and hyaluronidase (53 875.6 Da), with two major peaks of the two most abundant molecules in the venom (melittin and PLA2) (Figure 2C). Separately, the compound spectra analysis of peak 1/HPLC showed the presence of a molecular mass of 2027.35 Da (1013.5 + 2 H^+^) and that of peak 3/HPLC revealed a mass of 18 964 Da (1896.4 + 10 H^+^), while the analysis of peak 6/HPLC revealed a molecular mass of 2 846.4 Da (1423.2 + 2 H^+^) (see Figure S1).

### 2.2. Separation of Crude Venom Compounds by SPE

**Figure 3:**
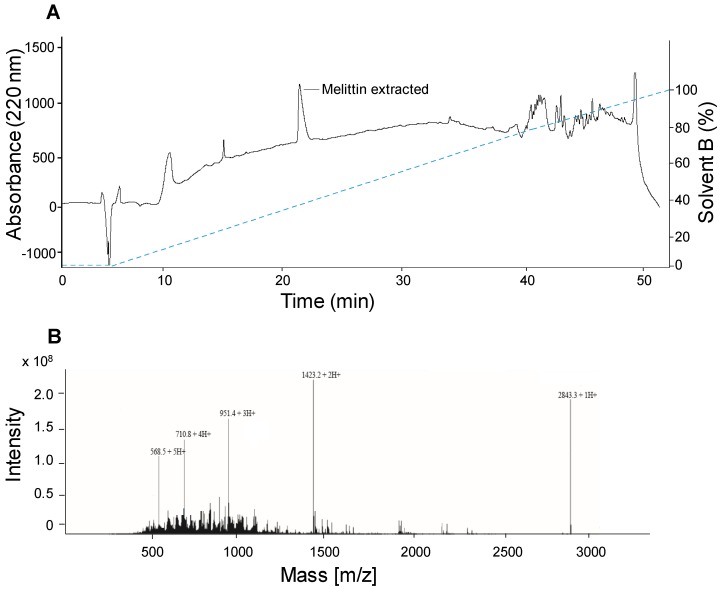
Isolation of melittin from the venom of *A. mellifera syriaca*. (**A**) HPLC profile of the melittin-containing fraction as a function of time and increasing % of acetonitrile solvent. (**B**) MS profile of the same fraction.

In order to separate and purify the different components of *A. mellifera syriaca* venom, we chose to use the SPE technique which allows the concentration of target compounds within the venom. It was operated with a C18 Cartridge by applying the same elution buffers of those used for HPLC. Five fractions were eluted, and each one could contain one or more molecules. These fractions were then analyzed with HPLC and the results obtained showed that the F4 fraction revealed a single peak in HPLC corresponding to the expected molecule—i.e melittin—according to the analysis with ESI-MS (Figure 3). This result suggests that SPE may be a relevant method for the separation of BV components and could be further used routinely in analytical toxicology.

### 2.3. Antibacterial Activity

**Figure 4:**
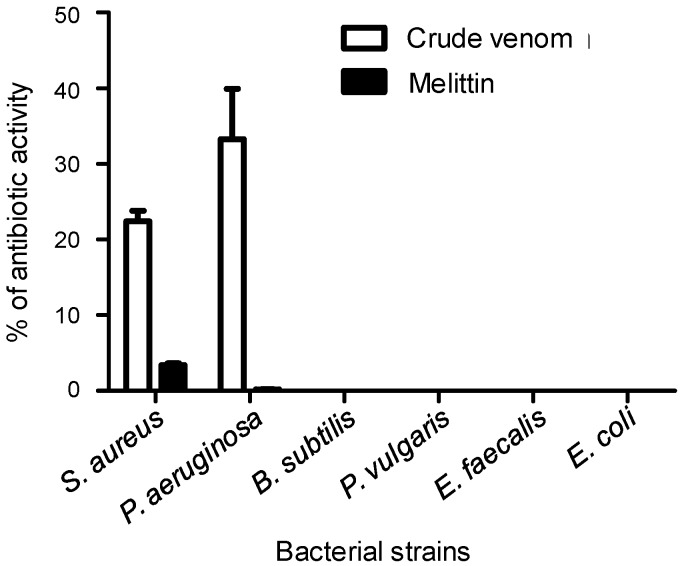
Antibacterial activity of *A. mellifera syriaca* venom. Growth inhibition of different bacterial strains by the venom as a percentage of the total inhibition by specific antibiotics (used as positive controls) is shown. H_2_O was used as a negative control. Crude venom inhibits bacterial growth of *S. aureus* and *P. aeruginosa.* Data are expressed as mean ± SD.

BVs are known to exert antibacterial effects [3]. We then decided to challenge the venom of *A. mellifera syriaca* against different bacterial strains, namely *Pseudomonas aeruginosa*, *Staphylococcus aureus*, *Bacillus subtilis*, *Proteus vulgaris*, *Enterococcus faecalis*, and *Escherichia coli*. At a concentration of 50 µg/mL, it exhibits antibacterial activity against *P. aeruginosa* and *S. aureus* strains only, with 38% and 21.4% inhibitions respectively. No activity (very low or not significant) was observed against other strains studied, such as *Enterococcus faecalis*. To gain further insight into this activity, we decided to assess the putative antibacterial capacity of melittin (1 mg/mL). No or weak activity of melittin was observed on all strains (Figure 4). 

### 2.4. Hemolytic Activity

**Figure 5:**
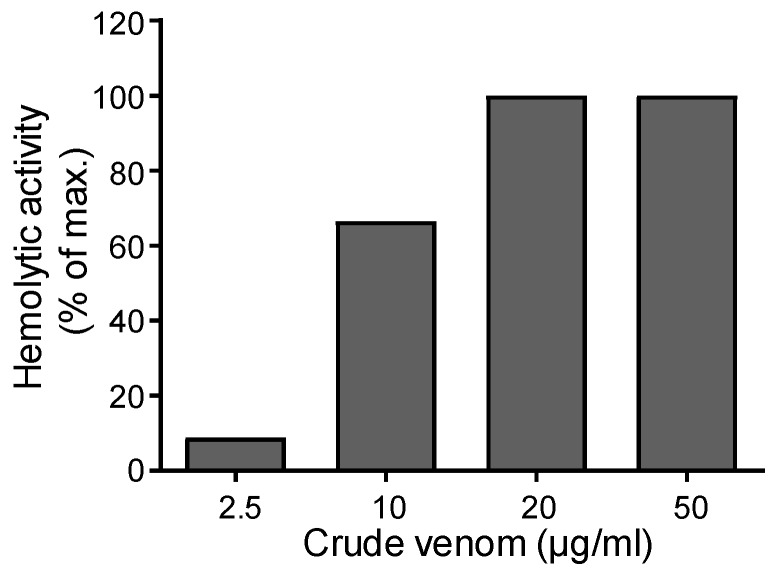
Hemolytic activity of *A. mellifera syriaca* venom. Different concentrations of venom (2.5–50 µg/mL) were used and hemolysis was quantified as a % of the maximal activity induced by H_2_O. A dose-response effect was observed and a plateau was reached from 20 µg/mL.

In order to test the hemolytic activity of *A. mellifera syriaca* venom, a suspension of red blood cells (RBCs) was subjected to different concentrations of crude venom (2.5; 10; 20; 50; 100; 150; 200; 300 and 500 µg/mL). After incubation, the absorbance of the different supernatants was read at 540 nm. The values obtained determined the percentage of hemolysis in each supernatant as a function of the concentration of the venom used, by comparing them with both positive (H_2_O) and negative controls (PBS). The venom of *A. mellifera syriaca* exhibits hemolytic activity: At a concentration of 2.5 µg/mL, 8.8% of hemolysis was observed, whereas, for a venom concentration of 10 µg/mL, 66.6% was recorded (Figure 5). A plateau was observed from a concentration of 20 µg/mL of the crude venom (100% hemolytic activity). These data suggest that *A. mellifera syriaca* venom harbors toxic components—namely melittin and PLA2—that act on the cell membrane of RBCs and induce their lysis.

### 2.5. Antioxidant Activity

**Figure 6:**
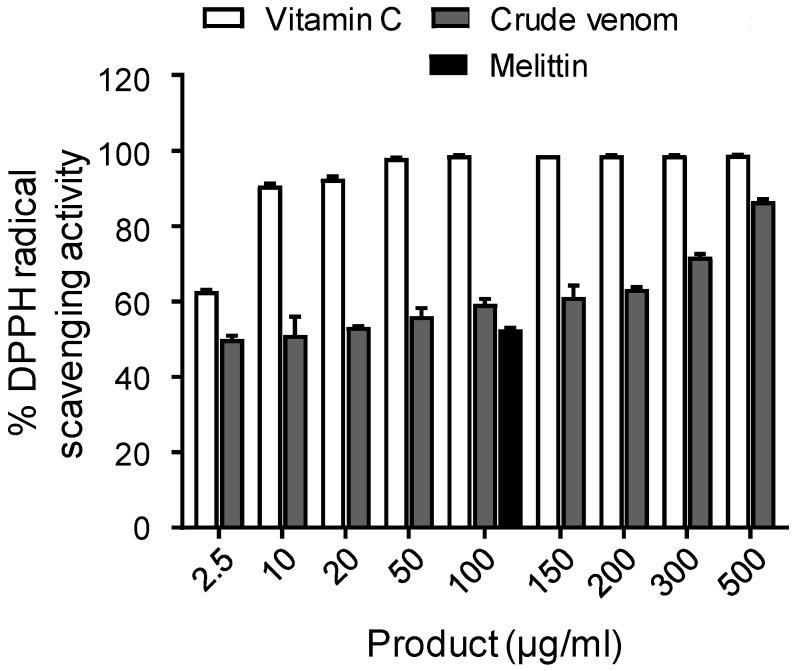
Antioxidant activity of *A. mellifera syriaca* venom. Different concentrations of venom (2.5–500 µg/mL) or melittin (100 µg/mL) were used, and absorbance was measured at 517 nm. Vitamin C was used as a positive control. The venom exhibits robust dose-dependent activity. Data are expressed as mean ± SD.

1, 1-diphenyl-2-picrylhydrazyl (DPPH) assay was used to evaluate the antioxidant activity. *A. mellifera syriaca* crude venom showed dose-dependent antioxidant activity (Figure 6). All concentrations used could exert an antioxidant action at a level close or weaker than vitamin C, used as a positive control. In fact, from 2.5–200 μg/mL, the percentage of DPPH radicals scavenging activity varies in the range of 50–65%. This value is increased at 71.9% for a concentration of 300 μg/mL, and reaches a maximum of 86.6% for the highest concentration used (500 µg/mL). Melittin was also tested. At a concentration of 100 μg/mL melittin, 52.5% of the scavenging activity was recorded.

### 2.6. PLA2 Activity

**Figure 7:**
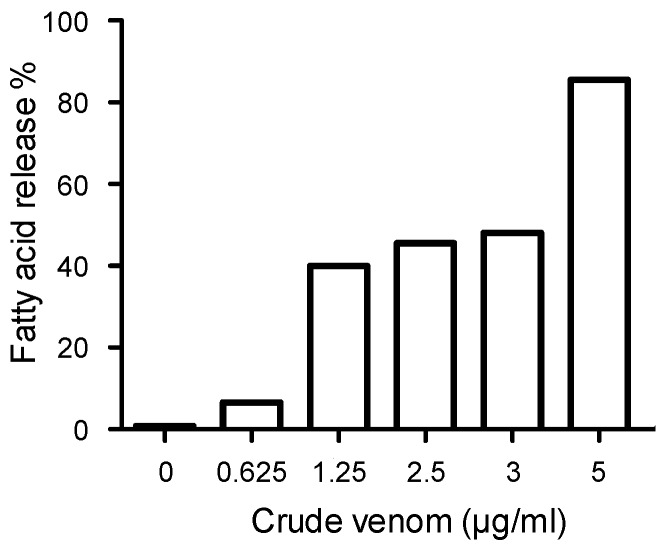
Effect of *A. mellifera syriaca* venom on fatty acid release. Phosphatidylcholine (PC) was subjected to increasing concentrations of the venom (0.625–5 µg/mL). Fatty acid release was measured as described in the methods. The venom exerts PLA2 activity.

BV produce inflammatory processes that lead to deep activation of nociceptors, thus inducing pain [16]. In snake venoms, it is well known that PLA2s are toxins which mediate pro-inflammatory activities. Indeed, they catalyze the hydrolysis of the sn-2 ester bond of membrane phospholipids, including phosphatidylcholine [17]. This phospholipid release favors the formation of arachidonic acid [18]. In the physiological context of inflammation, arachidonate is the molecular basis for the release of prostanglandins. As BV contain PLA2, we next challenged the crude venom and extracted melittin for their enzymatic activity to release free fatty acids, among which arachidonic acid is included. This test shows that crude venom has significant PLA2 activity (Figure 7). In fact, for a concentration of 5 μg/mL, 86.42% fatty acid release was observed. As expected, no effect was recorded for the melittin (100 µg/mL) extracted from the venom (not shown).

### 2.7. Cytotoxic Activity on MCF-7 and 3T3 Cancer Cells

**Figure 8:**
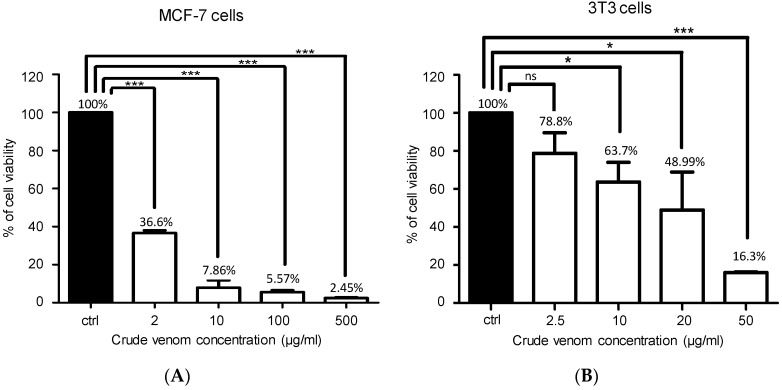
Cytotoxicity effect of *A. mellifera syriaca* venom on two different cancer cell lines. (**A**) Cytotoxicity activity of the venom on MCF-7 cells. (**B**) Cytotoxicity activity of the venom on 3T3 cells. Data are expressed as mean ± SD (*n* = 3–4). Unpaired t-test: ns (no significant), * *p* < 0.05; *** *p* < 0.001 when compared with the control. At 10 μg/mL, the crude venom showed a cytotoxic activity which is more significant against MCF-7 cancers cells.

BV has long been studied for their anti-tumoral and cytotoxic effects, which can be explained by their capacities to promote necrosis and/or apoptosis. The cytotoxic activity of *A. mellifera syriaca* venom was evaluated on two types of cancer cell lines: MCF-7 and 3T3. Results obtained show that this venom has dose-dependent antiproliferative activity against both (Figure 8). Nevertheless, this toxicity was stronger against MCF-7 than 3T3 cancer cells.

## 3. Discussion

In this study, we intended to separate and characterize the components of *A. mellifera syriaca* venom, with the aim characterizing its chemical and biological properties. The identification of various toxins correlates with previous studies demonstrating their presence in BV [2,19,20]. HPLC with gradient elution is usually used for the separation of venom components like proteins and peptides. These molecules can then be identified and characterized using ESI-MS. In fact, ESI has been a workhorse for the MS analysis of proteins and peptides [21]. In our study, LC-ESI-MS analysis of crude venom showed that melittin was the most abundant compound, followed by PLA2, as it has been described in BVs [2]. SPE is an original approach that reduces solvent exposure and extraction time and is also used to gain high recoveries [22]. SPE allowed us to purify and obtain high quantities of the different components of *A. mellifera syriaca* venom, especially melittin. 

The venom was then challenged in various assays to understand its biological activities. The antimicrobial effect against Gram+ and Gram- bacterial strains is in good accordance with previous studies describing the toxicity of *A. mellifera* venom [3]. It has been suggested that the toxicity of BV against bacteria is due in part to the presence of PLA2 and melittin [3,23]. In fact, our results showed that the antibacterial activity of *A. mellifera syriaca* crude venom was significant against some strains, mainly *S. aureus* and *P. aeruginosa*; however, at 1 mg/mL of purified melittin, activity was observed against *S. aureus* and not for the others bacterial strains. This confirms previous data showing that melittin was not effective against *P. aeruginosa* strains, but has a specific effect against *S. aureus* [24]. It has been reported that BV melittin is more active against Gram+ than Gram- bacteria, which suggests that PLA2 is the element responsible for the antibacterial activity observed on *P. aeruginosa* and *S. aureus* strains by causing a cleavage of membrane phospholipids and pore formation in the membrane followed by cell lysis. Alternatively, this activity may be produced through the synergetic action of melittin and PLA2 [3,23]. However, it would be necessary to challenge the antibacterial activity of *A. mellifera syriaca* PLA2 alone or in combination with melittin to confirm the origin of this toxicity against bacteria.

As shown in the past, melittin and PLA2 are together responsible for RBC lysis [25]. In fact, melittin, which is already known for its lytic activity, is considered to be the main cause of hemolysis. Melittin activates PLA2, in which catalytic activity causes the cleavage of the phospholipid bilayers, thus releasing lysophospholipids which become very active at the level of the membrane causing the destruction of RBCs [26]. Moreover, it has been reported that PLA2 purified from BV did not cause the lysis of RBCs, but when venom-purified melittin was added to the solution, hemolysis was observed [27]. As for the antioxidant effect of *A. mellifera syriaca* venom, our results are in accordance with previous studies on *Apis mellifera* venom reporting an antioxidant activity of BV extracts which inhibits the production of DPPH in a dose-dependent manner. Some data suggest that melittin alone exerts very poor antioxidant activity compared to BV extracts and this might be due to the influence of other venom components [2,28]. As for the pro-inflammatory effect of BV, it is due to PLA2, which has a phospholipid cleaving function: It releases arachidonic acid, a precursor of eicosanoids, which are robust inflammation mediators [29]. Moreover, melittin could play an indirect role by activating PLA2 which exerts its pro-inflammatory activity by increasing the secretion of chemical mediators such as pro-inflammatory cytokines [30].

Finally, the difference in BV cytotoxic activity against MCF-7 and 3T3 may be due to their specific membrane receptors. Previous studies demonstrated that BV components including melittin, apamin, and PLA2 exert anti-tumor activities against various types of cancer cell lines like mammary, renal, prostatic, and leukemic cells [31]. Moreover, previous data showed that BV acts as an anti-cancer agent through apoptosis, necrosis, and lysis induction of tumor cells via the activation of several signaling pathways involving a Bcl-2 protein, caspase 3, in synovial fibroblasts [32]. For instance, the antitumor effect of melittin is caused by the suppression of the production of matrix metalloproteinase: MMP-9 inhibition is correlated to the invasion inhibition of MCF-7 cells and the inhibition of caspase activity [33]. So, melittin derived from *A. mellifera syriaca* venom and/or even the crude venom may inhibit the proliferation of cancer cells and favor their apoptosis.

## 4. Conclusions

We characterized for the first time the *A. mellifera syriaca* venom. Its chemical composition reveals the presence of molecules already known in BVs such as apamin, melittin, PLA2, MCD-peptide, and hyaluronidase. MS analysis discloses unidentified experimental molecular masses, which may correspond to novel molecules with potential therapeutic interests. Several biological activities of *A. mellifera syriaca* crude venom were evaluated in vitro and the results obtained showed that this venom was able to inhibit the growth of certain bacterial strains that develop antibiotic resistance. The most significant result obtained in this work is its anti-tumoral effects, which revealed an antiproliferative action against MCF-7 and 3T3 cancer cells, making this BV a good natural precursor for the design of novel anticancer drugs. Paradoxical biological activities have emerged from this study, but this is a common feature in animal venoms. The next step will identify unknown components which could exhibit novel pharmacological properties.

## 5. Materials and Methods

### 5.1. Materials

#### 5.1.1. Bees

Healthy hives of local strains (*Apis mellifera syriaca*) were selected. The apiary was located in Ramlieh, Aley (Lebanon). The forage there is mainly from wild plantations and the flowers were fully blooming. 

#### 5.1.2. Venom

The venom was collected from healthy colonies of local *A. mellifera syriaca* strains. There was sufficient pollen in nature and in the hives (two frames of pollen in each colony). The collection was locally made following the standard electroshock method [34] and was installed at the top of the hive. When the wires were electrified and a mild shock was applied to the bees, they covered the surface of the wired glass plate and stung the surface of the glass plate in response to the electrical stimulation. Secreted venom from bee sting dried rapidly when exposed to the air. Dried venom was scraped off with a sharp scalpel and transferred to the laboratory and was stored at a temperature of −20 °C until further analysis. Extraction was made for 15–20 min on each colony and was repeated twice every 2 weeks. 

#### 5.1.3. Reagents

Acetonitrile (Acn), trifluoroacetic acid (TFA), phosphatidylcholine (PC), triton, dibasic sodium phosphate (Na2HPO4), monopotassium phosphate (KH2PO4), dimethylsulfoxide (DMSO), “Dulbecco’s Modified Eagle’s Medium” culture medium (DMEM, which contain 4500 mg/L glucose, L-glutamine, and sodium bicarbonate, without sodium pyruvate), the MTT kit, and vitamin C were purchased from Sigma Aldrich (Ibra Hadad, Beirut, Lebanon). The bacterial strains were provided by the microbiology laboratory of the Faculty of Public Health 3 of the Lebanese University in Tripoli.

### 5.2. Methods

#### 5.2.1. Chemical Characterization of The Crude Venom by LC-ESI-MS

Chromatographic separation was carried out using a Discovery^®^ HS C18 25 cm × 4.6 mm, 5 μm column. 5.5 mg of freeze-dried crude venom was dissolved in 1 mL of ultrapure water, then this amount was filtered using a syringe filter. 100 μL of the solution was injected into the HPLC. The collection process requires an elution gradient of 0–40% acetonitrile for 80 min at a flow rate of 1 mL/min, and a UV detector at 220 nm to separate the different components of the venom. The elution gradient used is composed of two eluents: Eluent A (0.1% TFA in water), and eluent B (0.1% TFA in acetonitrile). The fractions obtained and collected by HPLC were subjected to an ESI-MS analysis in order to identify and characterize the components of these fractions. This analysis was carried out in a scanning mode between 100 and 3000 m/z, following the same elution gradient conditions used for HPLC analysis, and similarly, the absorbance was measured at 220 nm. Data acquisition was recorded with the HyStar ™ and Esquire data system.

#### 5.2.2. Separation of Crude Venom Compounds using Solid Phase Extraction (SPE)

SPE of crude venom was performed using a C18 Cartridge. Also, 10 mg of *A. mellifera syriaca* venom was dissolved in 5 mL of ultrapure water, then this solution was filtered in a syringe filter. Two eluents were used: Buffer A (0.1% TFA in H_2_O) and buffer B (0.1% TFA in acetonitrile). Different % of elution were used to extract the compounds of the venom and these different elution gradients were chosen on the basis of HPLC data relating to the elution of each compound from *A. mellifera syriaca* venom. This indicates at what percentage of elution (eluent A: H_2_O and eluent B: Acetonitrile) each compound appears as. The first step of the SPE technique is the conditioning of the cartridge made by 100% acetonitrile to hydrate the silica. After this step, 100% H_2_O was added through the column, and the column was loaded with the venom sample (dissolved in H_2_O). After loading the sample, the various eluents were applied to the column, and the different fractions corresponding to the different eluents were collected and analyzed using HPLC in order to observe the quality of their contents and to evaluate their purity. 

#### 5.2.3. Antibacterial Activity

Ten mg of freeze-dried venom of *A. mellifera syriaca* were dissolved in 200 μL of ultrapure water. Similarly, a purified melittin solution from *A. mellifera syriaca* venom with an initial concentration of 1 mg/mL was tested as a standard. Crude venom and melittin were tested against six bacterial strains using sensitivity tests onto diffusion discs [35,36]. The first step of the procedure consists in the enrichment of the bacteria. Thus, the peptone water was prepared in tubes and then autoclaved at 121 °C for 15 min, then using a sterile loop, the bacteria were put in peptone water and mixed. The tubes containing bacteria were incubated at 37 °C for 24 h. Bacterial strains were then seeded on Petri dishes using a sterile loop and incubated at 37 °C for 24 h. Finally, six tubes were prepared (each one containing 3 mL of sterile water); their contents were poured into dishes containing Mueller Hinton medium, then the suspension was spread over the entire surface of the agar, and the excess was removed. After drying, the sterile filter paper discs were placed on the Petri dishes with sterile forceps. A volume of 10 μL/disc of the corresponding solution was added. Finally, the Petri dishes were incubated for 24 h at 37 °C. The area of inhibition was measured using a caliper. The antibacterial test was performed in duplicate. A specific antibiotic was used for each strain as a positive control (maximal activity) and H_2_O as a negative control. Data show the % of maximal effect.

#### 5.2.4. Hemolytic Activity

Hemolytic effect of *A. mellifera syriaca* crude venom was performed using human red blood cells (RBCs) [37]. Fresh blood was collected from healthy volunteers in EDTA tubes and centrifuged at 3000 rpm for 5 min. The supernatant containing serum and white blood cells were removed. The RBC pellet was washed three times with PBS and centrifuged each time at 3000 rpm for 5 min. A suspension of pure RBC was obtained. From this suspension, a volume of 100 μL was taken in each tube and treated with the venom at different concentrations from an initial stock solution (5 mg/mL in PBS). Two control tubes were prepared: One is considered as a positive control, which contains RBCs and distilled H_2_O, while the other corresponds to the negative control containing RBCs and PBS. All tubes were incubated at 4 °C for 30 min and then centrifuged at 3000 rpm for 5 min. Then, the absorbance of the supernatant was measured at 540 nm, and the absorbance values obtained determined the percentage of hemolysis in each tube. 

The hemolysis was calculated according to the following formula (where A designates Absorbance) [37]:

Hemolysis (%) = [(A _Tube_ − A _Negative Control_)/A _Positive Control_] × 100

#### 5.2.5. Antioxidant Activity Assay

The antioxidant activity was evaluated by applying the free radical scavenging method using DPPH (2,2-diphenyl-1-picrylhydrazyl) [2]. Five mg of lyophilized crude venom was dissolved in 1 mL of ultrapure water, and several samples (5 mg/mL) were prepared to test the antioxidant activity. Melittin (1 mg/mL) purified from the venom was also tested. Vitamin C was used as a positive control. A blank tube was performed for each dose of the venom as a negative control. Absorbance was measured at 517 nm before incubation using a spectrophotometer. The tubes were then incubated in the dark for 30 min, after which the optical density was measured at the same wavelength (517 nm). The antioxidant activity was performed in duplicate.

The % of DPPH radical scavenging activity was determined using the following formula (where A designates Absorbance):

DPPH radical scavenging activity (%) = [(A _DPPH_ − A _Echantillon_)/A _DPPH_] × 100

#### 5.2.6. Measurement of PLA2 Activity

To study the specific PLA2 activity of *A. mellifera syriaca* venom, phosphatidylcholine was used as a substrate [38]. The effect was measured by using a spectrophotometer based on pH change due to the release of free fatty acids from L-α-phosphatidylcholine [38]. First, a solution of the reaction medium was prepared as followed: 3.5 mM L-α-phosphatidylcholine (PC) ws placed in an Erlenmeyer flask into which 7 mM Triton X-100 (408 μL) was added. Also, distilled water was added to complete the volume to 50 ml. This solution was subjected to magnetic agitation for 1 h to promote the solubilization of the PC. Then, 10 mM CaCl2, 2H2O, 100 mM NaCl and 0.055 mM phenol red are added (pH 7.6). Finally, distilled water was added to reach a final volume of 100 mL. Different concentrations of crude venom were prepared from an initial solution of 5 mg/mL (in distilled water). Each tube contained 1 mL of the PC solution and the volume of the specified concentrations of venom. The tube representing the negative control consisted only of the PC solution. Absorbance was measured at 558 nm before incubation, and after incubation of the tubes at 37 °C for 5 min using a spectrophotometer.

The percentage of in vitro PLA2 catalytic activity was calculated using the following formula (where A designates absorbance):

Fatty acid release (%) = [A_negative control_ − (A_sample_/A_negative control_)] × 100

#### 5.2.7. Cytotoxic Activity Assay on MCF-7 and 3T3 Cancer Cells

The venom cytotoxicity was investigated using the MTT viability test [39]. An initial solution of 5 mg/mL of crude venom was prepared and this solution was filtered using a syringe filter. Cells were cultured in DMEM culture medium, until confluence. A plate of 24 wells was used in each well 1 mL of each prepared solution at different concentration was deposited. For each solution, triplicate copies were made for the MCF-7 cells, and four copies were made for 3T3 cells. This plate was incubated at 37 °C for 24 h. After incubation, a volume of 10 μL MTT was added in each well. This step was performed in dark, as MTT is photosensitive. The plate was stirred and then incubated at 37 °C for 1h. The medium was then removed and 1 mL DMSO was added to each well to solubilize Formazan crystals. Absorbance quantification was read at 560 nm.

#### 5.2.8. Statistical Analysis

Results were expressed as the mean ± standard deviation (SD). Statistical significance between different samples was analyzed using a two-tailed unpaired t-test. Statistical significance was defined as * *p* < 0.05, ** *p* < 0.01 and *** *p* < 0.001. This analysis was carried out using GraphPad Prism 7.02 (GraphPad Software, San Diego, USA).

## Supplementary Materials

The following are available online at https://www.mdpi.com/2072-6651/11/4/191/s1, Table S1: Experimental molecular weights (Da) obtained by ESI-MS analysis of the *A. mellifera syriaca* venom eluted from HPLC, Figure S1: High resolution mass spectra of melittin, apamin and PLA2 obtained from the venom of *A. mellifera syriaca*.
