# Supplementary material for: First Characterization of The Venom from Apis mellifera syriaca, A Honeybee from The Middle East Region
Source: Toxins (Basel). 2019 Mar 30;11(4):191. doi: 10.3390/toxins11040191 (PMC6521255; doi:10.3390/toxins11040191)
Supplement: Supplementary file 1 [file toxins-11-00191-s001.pdf]

Supplementary Materials: First Characterization of The Venom from *Apis mellifera syriaca*, A Honeybee from The Middle East Region

Jacinthe Frangieh, Yahya Salma, Katia Haddad, Cesar Mattei, Christian Legros, Ziad Fajloun and Dany El Obeid

Table S1. Experimental molecular weights (Da) obtained by ESI-MS analysis of the *A. mellifera syriaca* venom eluted from HPLC. In red, molecular weight of compounds already described in BV (apamin, melittin, PLA2, hyaluronidase and MCD peptide). Shown are the increasing MWs (top to bottom and left to right). See text for details.

487.2 (*Mr* + *1H*+)	994.2 (*Mr* + *1H*+)	643.8 (*Mr* + *2H*+)	1000.9 (*Mr* + *2H*+)	**1422.7 (*Mr* + *2H*+)**
590.2 (*Mr* + *1H*+)	1097.2 (*Mr* + *1H*+)	1323.5 (*Mr* + *1H*+)	1001.8 (*Mr* + *2H*+)	1493.4 (*Mr* + *2H*+)
608.9 (*Mr* + *1H*+)	1105.8 (*Mr* + *1H*+)	749.5 (*Mr* + *2H*+)	**1013.5 (*Mr* + *2H*+)**	1578.5 (*Mr* + *2H*+)
697.8 (*Mr* + *1H*+)	558.2 (*Mr* + *2H*+)	778.3 (*Mr* + *2H*+)	1169.1 (*Mr* + *2H*+)	1587 (*Mr* + *2H*+)
884.8 (*Mr* + *1H*+)	1170.9 (*Mr* + *1H*+)	792 (*Mr* + *2H*+)	1225.2 (*Mr* + *2H*+)	1896.4 (*Mr* + *2H*+)
898.5 (*Mr* + *1H*+)	591.4 (*Mr* + *2H*+)	802.6 (*Mr* + *2H*+)	1256.2 (*Mr* + *2H*+)	951.4 (*Mr* + *12H*+)
943.7 (*Mr* + *1H*+)	587.4 (*Mr* + *2H*+)	1640 (*Mr* + *1H*+)	**1299.9 (*Mr* + *2H*+)**	**1896.4 (*Mr* + *10H*+)**
958.2 (*Mr* + *1H*+)	609.5 (*Mr* + *2H*+)	995 (*Mr* + *2H*+)	1353 (*Mr* + *2H*+)	**2565.5 (*Mr* + *21H*+)**

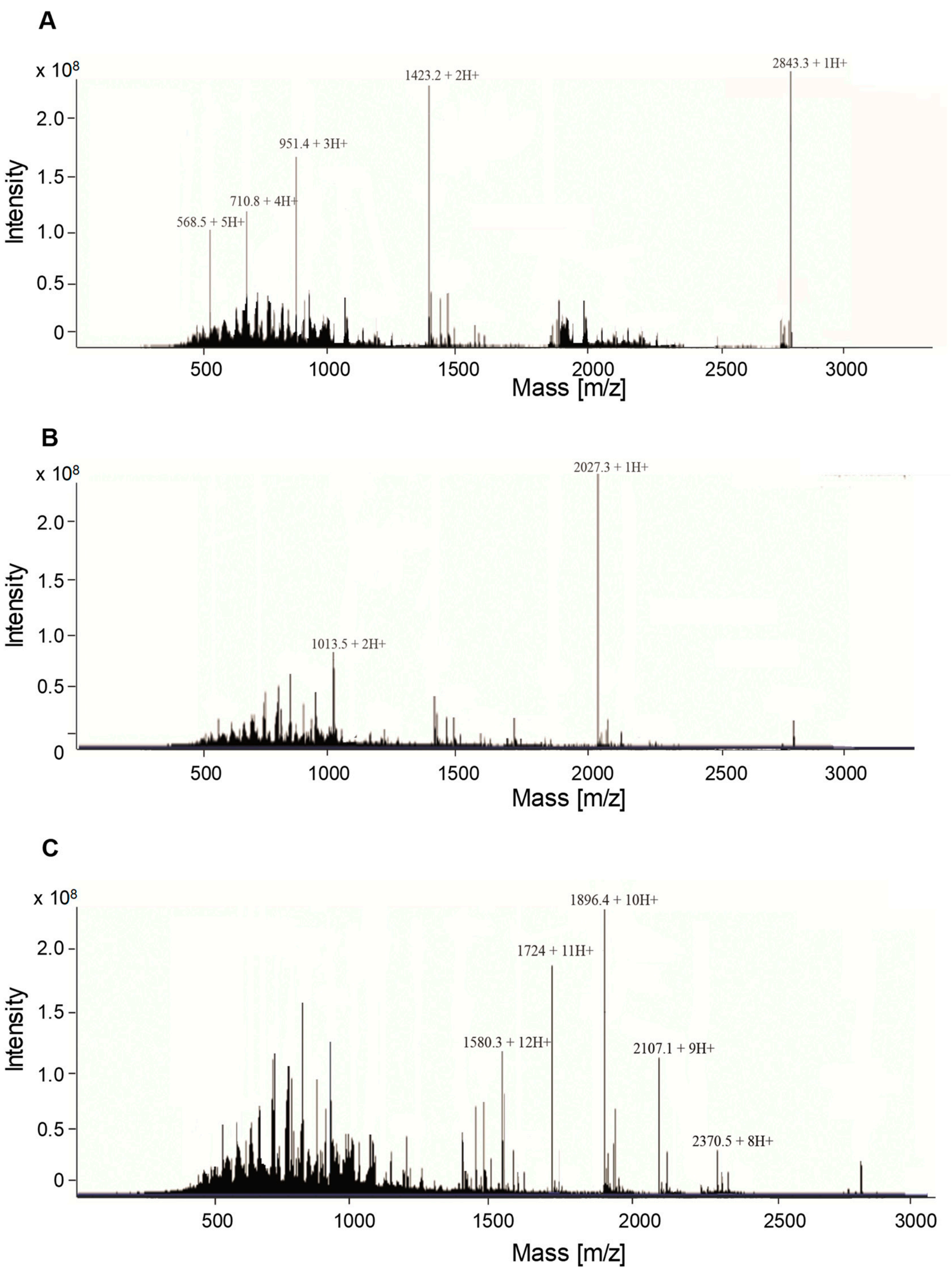

Figure S1. High resolution mass spectra of melittin, apamin and PLA2 obtained from the venom of *A. mellifera syriaca*. (**A**) mellitin. (**B**) apamin. (**C**) PLA2.
